# Inventory of Survey Instruments for Monitoring Antimicrobial Use in Primary Care Settings in Low- and Middle-Income Countries: A Narrative Review

**DOI:** 10.3390/antibiotics14111159

**Published:** 2025-11-15

**Authors:** Verica Ivanovska, Tracey-Lea Laba, Renly Lim, Anita Kotwani, Arno Muller, Martina Escher, Benedikt Huttner, Elizabeth Roughead

**Affiliations:** 1 Antimicrobial Resistance Department, World Health Organization, 1211 Geneva, Switzerland; amuller@who.int (A.M.); escherm@who.int (M.E.); bhuttner@who.int (B.H.); 2 Centre for Health Economics Research and Evaluation, University of Technology, Sydney 2007, Australia; proxylaba@gmail.com; 3 Clinical and Health Sciences, University of South Australia, Adelaide 5000, Australia; renly.lim@unisa.edu.au (R.L.); libby.roughead@unisa.edu.au (E.R.); 4 Vallabhbhai Patel Chest Institute, University of Delhi, Delhi 110007, India; anitakotwani@gmail.com

**Keywords:** antimicrobial, use, outpatients, primary care, AWaRe, low- and middle-income countries (LMICs)

## Abstract

**Background:** Over 80–90% of antimicrobial use occurs in primary health care, underscoring the need for specific data from this sector to inform practices and interventions to improve antimicrobial use. This study aimed to identify a wide range of research instruments in primary health care and qualitatively describe their structure, scope, and content. **Methods:** For the narrative review, we reviewed Medline (inception–November 2023) and agency/network websites to identify surveys on antimicrobial use prevalence in LMIC primary care. We applied no language restrictions and extracted survey instruments from publications or requested them from authors when unavailable. **Results:** We identified 450 studies and extracted 42 survey instruments issued between 1993 and 2023, all but one post-2000. These covered both multi-country (16.7%) and country-specific implementations across all WHO regions. Sampling units included households/consumers (24/42, 57.2%), health professionals (14/42, 33.3%), drug sellers (3/42, 7.1%), and bulk sales data (1/42, 2.4%). Surveys typically captured antimicrobial type, prescription status, and reason for use; AWaRe classification was mentioned only once. We found 13 stand-alone protocols on antimicrobial use and 4 on general medicine use. **Conclusions:** We identified diverse tools for measuring antimicrobial use in LMICs, though many lacked protocols or analytic support. Surveys often focused solely on antibiotics, used paper-based methods, and rarely referenced the AWaRe classification. Future efforts should broaden the scope beyond antibiotics, leverage digital data systems, include implementation protocols and analytic tools, report standardized indicators, and adopt AWaRe-related variables as a core criterion to strengthen AMU monitoring in PHC.

## 1. Introduction

Antimicrobial stewardship (AMS) is one of the most important cornerstones of global efforts to reduce antimicrobial resistance (AMR) [1]. To be effective, AMS requires knowledge of antimicrobial use (AMU) so that interventions can be targeted to areas of need [1]. However, measuring AMU globally is challenging due to variations in health systems, health funding, and issues with data availability often due to the supply of antimicrobials from multiple sources. In low- and middle-income countries (LMICs), data collection is often constrained by labor-intensive, paper-based methods; limited resources; inadequate infrastructure; weak regulatory frameworks; and the need for capacity building. In contrast, many high-income countries (HICs) have long utilized electronic health data to monitor antimicrobial use. However, even in HICs, health systems may seek more detailed information, such as data on indication, which is not always systematically captured [2]. To strengthen national surveillance systems, the World Health Organization (WHO) established the Global Antimicrobial Resistance and Use Surveillance System (GLASS) in 2015. In 2020, GLASS included a dedicated module for antimicrobial use (GLASS-AMU), enabling countries to report standardized data on antimicrobial use. Under the GLASS-AMU methodology, data are reported at the national aggregated level using medicine-level (m-AMU) information on the types and quantities of antimicrobials used. However, m-AMU data lack clinical-level (c-AMU) details such as patient characteristics, diagnoses, treatment indications, and outcomes. Additionally, national m-AMU data are not disaggregated by sector (e.g., public vs. private) or by level of care (e.g., primary health care vs. hospitals). In many cases, countries are unable to collect disaggregated data due to limitations in their surveillance systems. As of 2025, 74 countries have submitted national aggregated m-AMU data to GLASS-AMU covering the period from 2016 to 2022, of which 39 are LMICs [3].

To further support appropriate antimicrobial use, the WHO also developed the WHO AWaRe system and antibiotic book [4]. AWaRe groups antibiotics into Access (narrow-spectrum, safer, and recommended as first-line for common infections), Watch (broader-spectrum, used for more severe or resistant infections), and Reserve (last-resort options for multidrug-resistant infections). AWaRe, as a recent global stewardship tool, now serves to track progress toward the UN General Assembly’s target of ensuring that at least 70% of antibiotic use comes from the Access group [5]. Data from the 2022 GLASS report show wide variation in total and AWaRe-specific antibiotic use globally [3]. More detailed, sector-specific data, particularly from primary care and hospital settings, are needed to guide targeted stewardship efforts.

This review focuses on obtaining sector-specific data on antimicrobial use in outpatient settings, also known as primary health care (PHC). These settings account for up to 90% of antimicrobial prescriptions, often for viral infections such as acute respiratory tract infections, where antibiotics are not needed [6,7]. While hospital stewardship is important, the volume of antibiotic use in PHC makes it a priority for intervention and monitoring. Yet measuring outpatient antimicrobial use can be challenging as it encompasses a wide range of settings and providers. PHC includes formal health facilities such as public health centers, private clinics, hospital outpatient units, emergency services, dentist clinics, pharmacies, and remote health outposts. In LMICs, PHC also extends to informal providers, including community health workers, unlicensed practitioners, corner shops, and street medicine vendors. Furthermore, self-medication with antibiotics obtained over the counter without a prescription is widespread and must be considered within the broader PHC landscape [8]. This inclusive definition of PHC is used throughout this review to reflect the realities of medicine use in LMICs. This complexity requires a nuanced and comprehensive approach to monitor medicine use. Methods can include (a) consumer-directed data collection (e.g., household surveys), (b) health professional surveys (e.g., dispensing or prescription surveys), and (c) aggregated medicine use datasets from procurement, bulk sales, or import records [9].

In 2017, researchers from the London School of Hygiene and Tropical Medicine (LSHTM) collated protocols and methods used to measure antimicrobial use in both humans and livestock, summarizing data collection methods, sampling strategies, and indicators [10]. However, the survey instruments themselves were not included, and the AWaRe classification, established by WHO only after 2017, was therefore not part of the study. So, to date, no review has examined the survey instruments used for monitoring antimicrobial use in PHC settings in LMICs. We collated and analyzed those survey instruments and assessed whether they incorporate the AWaRe classification. This review, along with our separate ongoing review focused on high-income countries, will inform a forthcoming WHO guidance document on monitoring antimicrobial use in PHC.

## 2. Methods

### 2.1. Design

We chose a narrative review design underpinned by a structured search of the published literature and a purposive search of the gray literature instead of a systematic review [11] to capture and describe as many survey instruments as possible, as the actual survey instruments are not always published in the academic press.

### 2.2. Definition

Our primary units of interest were data collection instruments (e.g., questionnaires, data collection forms, or full protocols) to measure antimicrobial use in primary care in LMICs. Data collection instruments were defined as instruments for measuring antimicrobial use, while protocols were considered to be methods for implementation of the data collection instruments.

The analysis included all LMICs from each of the six WHO regions: the African Region, the Region of the Americas, the Eastern Mediterranean Region, the European Region, the South-East Asia Region, and the Western Pacific Region. (See all inclusion criteria below).

The outpatient use referred to a range of diverse settings: public health centers, private clinics, hospital outpatient units, emergency services, dentist clinics, pharmacies, remote health outposts, informal providers (i.e., community health workers, unlicensed practitioners, corner shops, and street medicine vendors), and self-medication (i.e., over-the-counter use without a prescription).

### 2.3. Search Strategy

The previously mentioned LSHTM work [10], together with a 2020 systematic review on the prevalence of antimicrobial use in primary care settings in LMICs (which included 48 studies conducted between 2010 and 2019) [12], served as the starting point for our search. Similarly, we searched Medline without language restrictions from inception to November 2023 (Supplementary Materials). The search included terms and subject headings for the following concepts: LMICs’ medicine use, settings, methods, and indicators. Surveys in languages other than English were translated where possible. Our multi-pronged search strategy also included screening reference lists of identified papers, conducting purposive searches in PubMed and Google Scholar using author names from related studies, reviewing websites of international health organizations, and contacting experts in medicine use monitoring, identified through their collaboration with WHO and involvement in technical projects, to obtain information about unpublished instruments. 

### 2.4. Extraction of Instruments from Studies

Two reviewers (the second and the last author) reviewed in full studies where methods indicated that surveys, questionnaires, or audits had been used to measure antimicrobial use in primary care in LMICs. We determined whether the survey instrument or data collection form was included in the article or Supplementary Files. Where survey instruments were unavailable, we requested copies of the data collection instruments from the authors. 

### 2.5. Inclusion Criteria

We included survey instruments to measure antimicrobial use in primary health care in LMICs, comprising outpatient facilities such as primary health care clinics, ambulatory and outpatient departments of hospitals, and community pharmacies. The World Bank’s 2024 classification was used to determine LMIC status [13]. We excluded the following:

(a) Research undertaken in hospital inpatient settings, hospital entry surveys assessing medicine use prior to admission, or studies where inpatient and outpatient use could not be differentiated;
(b) Focus group discussions that only measured knowledge of or attitudes towards antimicrobial use or resistance, or used hypothetical clinical scenarios or simulated patients;
(c) Tools where the primary purpose was to measure access to medicines or their prices.

### 2.6. Data Extraction

We identified all studies that included a survey instrument designed to measure antimicrobial use through a structured search strategy. Each study was treated as a single sampling unit and characterized using pre-specified categories across multiple dimensions [10]. For the target population, categories included consumers, physicians, pharmacists, drug sellers or informal health workers, and health agencies. Survey types were classified as household interviews, exit interviews, practice audits (retrospective or prospective), facility audits, import/procurement/sales data analysis, or electronic health/claims data analysis. Population characterization was based on whether surveys targeted children only, adults only, or all age groups (as defined by the authors). Disease or symptom-specific surveys were categorized by focus on respiratory tract infections, fever, diarrhea, pneumonia, or urinary tract infections, as these represent the most common symptoms and infectious conditions encountered in outpatients in LMICs and are key drivers of antibiotic prescribing in primary health care [14]. Survey sites were classified as household, health facility, warehouse, or national agency. We also recorded the country of implementation and used pre-set criteria to describe each instrument’s structure, including the number of questions, item format (closed or open-ended), and response type (closed or structured).

For each survey, we identified the main reported measure of antimicrobial use, along with other collected information such as antimicrobial type, indication, treatment duration, source of supply (prescription or over the counter), cost or price, and source of recommendation for use. All identified surveys were documented with published links included in the results tables and unpublished instruments listed in the Supplementary File.

### 2.7. Data Analysis

To support the qualitative analysis, we organized the abstracted data into standardized summary tables, each grouping studies by survey type to enable structured comparison across instruments. We aimed to examine how instruments captured antimicrobial use in PHC settings in LMICs, focusing on aspects such as the target population, disease focus, implementation site, country, and reported measures of use. This approach was intended to identify patterns in instrument design and scope, highlight variation in the types of data collected (e.g., prevalence, prescription status, and indication), and support a descriptive synthesis of strengths and limitations across tools.

## 3. Results

### 3.1. Search Results

**Figure 1:**
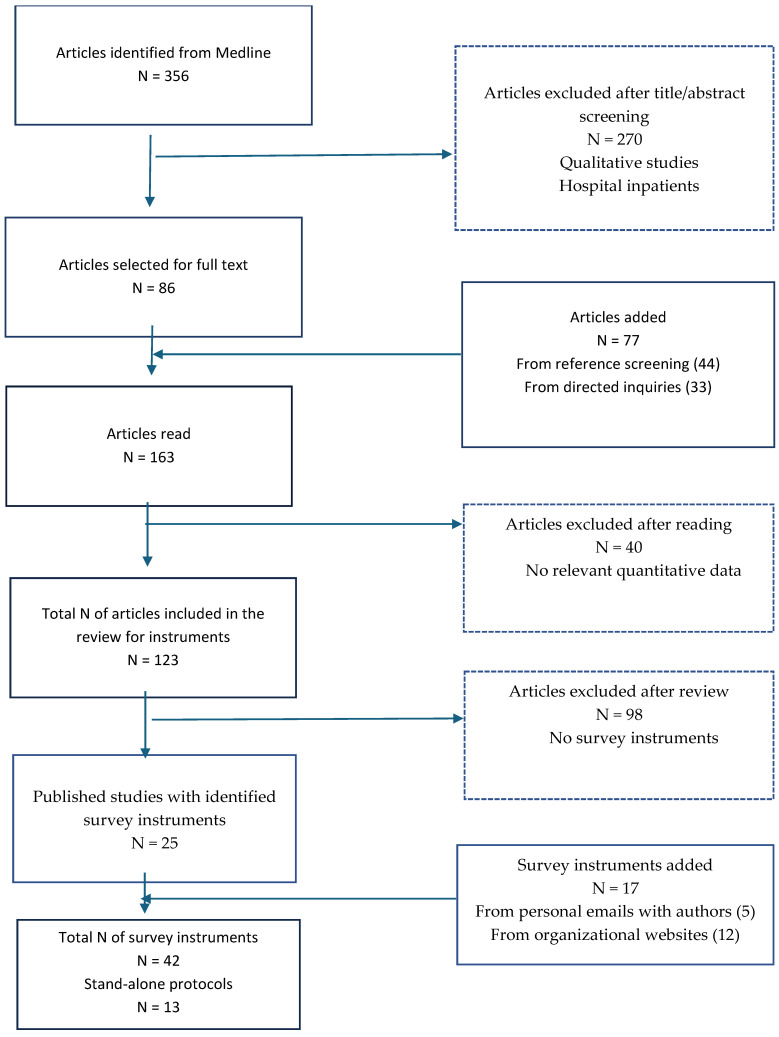
Flow diagram summary of the survey identification process.

We identified 450 studies, 356 from Medline and 94 from other sources, which were composed of reference screenings (N = 44), inquiries to authors (N = 33), and organizational websites (N = 17) (Figure 1). After the initial screening, qualitative and hospital inpatient studies were excluded. Full-text articles were reviewed, and studies lacking relevant quantitative results or survey instruments were removed. In total, 25 published studies with survey instruments were included in the analysis (Figure 1). We identified an additional 12 instruments from health organizations’ websites (Table 1) and five unpublished ones from authors for a total of 42 instruments (Figure 1). 

While searching for survey instruments, we found 13 protocols on medicine use surveys. Four of these were directly associated with identified survey instruments and part of the same studies. The remaining nine were independent and not linked to any specific instrument. These protocols provide comprehensive plans on how to employ surveys on the use of medicines, detailing the survey methodology, procedures, consent processes, interviewer training, data collection timelines, and other operational aspects. As these protocols were not part of the primary objective of our study, they are presented in the Supplementary Materials.

### 3.2. Overall Characteristics of the Survey Instruments 

**Figure 2:**
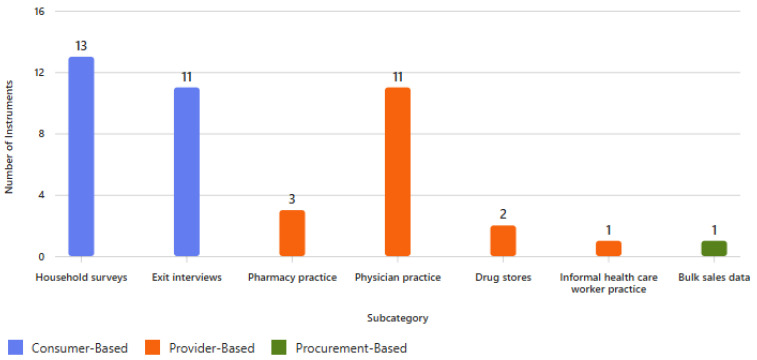
Number of identified survey instruments by method used.

As shown in Figure 2, survey methods were mostly consumer-based, especially household surveys (13/42, 31%) and exit interviews (11/42, 26.2%), followed by provider-based approaches like physician practice (11/42, 26.2%) and pharmacy practice (3/42, 7.1%). Table 2 shows that almost all studies with survey instruments (41/42, 97.6%) were conducted after the year 2000. Surveys were geographically diverse, with the WHO South-East Asian Region (8/42, 19%) most represented and equal contributions from the WHO African Region, the WHO Western Pacific Region, and multinational surveys conducted across multiple regions (each 7/42, 16.7%).

### 3.3. Consumer-Directed Surveys

#### 3.3.1. Household Surveys

We identified thirteen examples of household surveys [15,16,17,18,19,20,21,22,23,24,25,26,27] (Table 3). Five were generic surveys, not restricted by population or disease [15,16,17,18,27]. Four focused on children under five years or two years [19], and three were disease- or symptom-specific [23,24,25] (two disease-focused in children), with the targeted infections including respiratory, gastric, and urinary conditions. One survey included a One Health focus [26], encompassing questions about human, animal, and agricultural antimicrobial use. One was generic to any medicine use (i.e., not specific to antimicrobials) [17].

Most household surveys (9/13, 69.2%) were based upon a self-reported period prevalence of antimicrobial use during a defined recall period. Measures related to clinical context included type of medicine (10/13, 76.9%), indication (9/13, 69.2%), treatment duration (6/13, 46.2%), prescription status (5/13, 38.5%), and source of recommendation for use (5/13, 38.5%). Measures related to access and patient behavior included source of supply (9/13, 69.2%), proportion taken (2/13, 15.4%), proportion saved for future use (2/13, 15.4%), and proportion shared (1/13, 7.7%). No study reported AWaRe-related measures.

#### 3.3.2. Exit Interviews

We identified eleven exit interview tools [16,28,29,30,31,32,33,34,35,36] (Table 4). Four were disease-focused, with a focus on gastrointestinal [34,35] or respiratory illness [33,36]. All but allowed point prevalence of antimicrobial use to be measured. Other reported measures included the type of medicine; its indication, dose, and duration; and whether it was purchased over the counter or dispensed. A single study from 2017 stratified the antibiotics by AWaRe categories [29].

### 3.4. Health Facility Surveys and Data Collection Forms

#### 3.4.1. Pharmacy Audit Surveys

We identified three pharmacy practice audit surveys [37,38,39] (Table 5). Two were antimicrobial-focused [37,38], recording the antimicrobials dispensed and the indication for use, while the other was disease-focused, recording treatments provided for diarrhea [39]. These surveys generally provided a measure of the type of antimicrobial supplied as a proportion of all antimicrobials supplied, with the disease-focused survey recording the proportion of encounters during which an antimicrobial was supplied.

One of the antimicrobial-based surveys used three forms dependent on the source of the antimicrobial request, (i.e., one form for a prescription order, one form for pharmacist-initiated supply (dispensing without a prescription), and one form for a patient-initiated request (self-medication)) [37]. The other antimicrobial-based surveys captured the source of antimicrobial request (i.e., prescription or not) using a question within the form [38,39]. Other reported measures included the type of medicine and its indication, dose, duration, and cost. No study reported AWaRe-related measures.

#### 3.4.2. Physician/Primary Care-Directed Surveys

We identified eleven physician-directed surveys that enabled audits of practice [40,41,42,43,44,45,46,47,48,49,50] (Table 6). Three were generic case report forms that allowed recording of any problem managed or reason for encounter, along with associated treatments or investigations, using unstructured text. Three were disease-focused [48,50], focusing on respiratory tract infections, one of which provide a fully structured audit form [50]. These tools provide a measure of the proportion of encounters during which an antimicrobial was prescribed and, frequently, the indication for use and duration of antibiotic treatment. No study reported AWaRe-related measures.

#### 3.4.3. Corner Store/Drug Store- and Informal Health Care Worker-Directed Surveys

The only surveys we identified to measure antimicrobial use in corner stores or drug stores were surveys measuring whether antimicrobials were stocked and provided information on the types of antimicrobials sold during a defined recall period but not the quantities dispensed [51,52] (Table 7). This is likely due to the lack of documentation, as sellers are usually not required to record dispensed medicines.

We identified one survey targeting informal health care workers. Provision of a customized duplicate prescription pad form to participants overcame the challenge of informal care workers usually not keeping documentation of supplies [51]. Other reported measures included the type of medicine and its indication, dose, and duration. No study reported AWaRe-related measures.

### 3.5. Aggregated Data

#### Bulk Sales Data

Bulk sales data, if limited to the primary care sector, can provide insight into volume of use but not prevalence of use. One example of a standardized data collection form identified to support the calculation of volume of use using DDD/1000 inhabitants/day was used in South Africa for assessing antimicrobial use in both public and private sectors [52,53] (Table 8). No study reported AWaRe-related measures.

### 3.6. Stand-Alone Protocols

Along with the survey instruments, we identified 13 detailed and comprehensive protocols on how to conduct surveys on antimicrobial use in primary care in LMICs [16,26,27,31,38,40,46,54,56,57,58,59,60] (Table S1, Supplementary Materials). They outline detailed survey plans, including their methodology, consent, training, and data collection procedures. The majority (nine) were independent of the surveys reported in the previous section.

The protocols were either multi-country [16,27,31,38,46,60] or related to countries or WHO regions: Americas [27,31], Africa [54,56], Eastern Mediterranean [38], European [38], South-East Asia [57,59], and Western Pacific [26,40,58]. Most of the protocols were applicable to the whole population.

The data collection methods included in the protocols included household surveys, exit interviews, health facility practice audits, and analyses of sales data or electronic claims data. Two protocols from the published literature [26,59] and one from a government website employed a “One Health” model encompassing measurement of human, animal, and agricultural use in addition to capturing data on resistance levels. Two of the One Health protocols included household surveys as one type of data collection method. These household surveys included questions beyond human use alone [26,59].

We also identified one protocol that was focused on the management of febrile illness in LMICs and used the drug bag method for data collection during a household interview [60]. This method involved participants sorting provided antimicrobials into piles based on familiarity, usage, and recent use during illness [61].

We identified four protocols, all published by the WHO, to measure medicine use [17,43,48,49] (Table S2, Supplementary Materials). These protocols did not specifically focus on antimicrobial use. All provided details on sampling sizes and methods, data collection methods, and data collection forms. There was also a related reference text on how to investigate medicine use published by Management Sciences for Health [62].

## 4. Discussion

We identified a range of survey tools and protocols that had been used to monitor antimicrobial use in LMICs. These were used for surveys targeting consumers, health facilities, and informal workforces, while one study examined bulk sales data for routine monitoring. The reported measures of use included: point prevalence or period prevalence, proportion of encounters with antimicrobial use, and volume of use measured by DDD per 1000 inhabitants per day (DID). Of the 18 studies published since 2017, only one reported AWaRe-related measures based on general practice records. This limited evidence cannot be used to assess antibiotic use quality or progress toward the UN target of 70% Access group consumption.

### 4.1. Consumer and Household Surveys

A variety of the household surveys and exit interviews identified allowed measurement of point or period prevalence of antimicrobial use. These surveys all relied on self-reporting. The major limitation of self-reporting is the limited accuracy of the recall of illness events [63]. Surveys using shorter recall periods (e.g., one-month or two-week period prevalence measures) [15,16,64] may provide more accurate estimates, as may surveys with more intensive data collection methods, such as those using illness diaries and biweekly visits [19,20]. These methods, however, may also require more resources or larger sample sizes. An advantage of consumer surveys is that they can explore whether antimicrobials were prescribed or self-medicated, their source and recommendation for use, and the percentage of medicines taken, shared, and saved for future use. Knowledge of which antimicrobials are used by consumers and under what circumstances can inform interventions to improve antimicrobial use by consumers. Studies have reported that consumer knowledge and awareness about the appropriate use of antibiotics are often limited [65].

### 4.2. Pharmacy, Physician, and Informal Practice Audits

Various survey tools and data collection instruments have been used to audit pharmacy and physician practices, as well as to examine antimicrobial sales in medicine stores and corner stores and by informal health workers. Two structured forms for physician practice audits were specific for upper respiratory tract infections. The pharmacy surveys provided insight into the types of antimicrobials supplied in pharmacies and whether they were on prescription or over the counter, as well as the reason for use. A limitation of the antimicrobial-focused forms is that they did not provide an overall measure of use, unless all encounters were recorded as the denominator. The two structured forms for medicine stores or corner stores only measured whether antimicrobials were present or not and the types of medicines sold; they did not provide quantitative measures of antimicrobial use. Although pharmacy practice audits used structured questions, this method of data collection also relied on text entry responses, particularly for medicine name entry, which has a high risk of error, resulting in missing data due to illegibility, incomplete information, or spelling errors. Structured response forms that include a list of key antimicrobials from the AWaRe classification [4] or electronic forms with drop-down menus for medicine selection could minimize such risk.

Most surveys had less than 20 items. However, we lacked information on the time required to complete the survey and the simplicity of use. The major limitation of the surveys identified was lack of structured responses. Surveys with closed questions and structured responses are the easiest to implement and analyze [63]. A limitation of all the pharmacy forms was that they all had some unstructured responses. Consideration could be given to adapting these tools, identifying the key set of questions with structured answers required, e.g., focusing on the main diagnoses in primary care and recommended treatment according to the WHO AWaRe antibiotic book and/or national guidelines [4].

### 4.3. Routinely Collected and Electronic Data Sources

Only one study analyzed existing routinely collected records, specifically bulk sales data. Enabling ongoing monitoring of antimicrobial use by developing electronic versions of the survey instruments linked to a central database and dashboard to support ongoing data capture and analysis may also be possible in some circumstances. For example, Namibia developed an integrated pharmaceutical management system that tracks stock and patient-level medicine use. Originally designed for antiretroviral therapy management, it includes electronic dispensing and stock control data, feeding into a national dashboard that highlights stock issues and treatment numbers [66]. An electronic data capture system has also been trialed in poor areas of Kenya with consumers using a health wallet on their mobile phones to start a claims process, with all data, including dispensing, clinic visits, and pathology tests, captured through a data exchange platform [33]. Electronic data capture for antimicrobial measurement has also been reported in China [67], Thailand [68], Ecuador [69], Burkina Faso [70], Hungary [71], Serbia [72], and Colombia [73,74]. Many LMICs self-report having some form of national monitoring system for the use of medicines, including South Africa [75], Iran [76], Malaysia [77], Thailand [69], and the Philippines [40].

### 4.4. Methodological Limitations of Existing Tools

We employed a narrative review approach to allow for an iterative and flexible exploration of the literature, which is better suited to capturing the breadth of available data across diverse sources and formats. A limitation of our study is that some survey instruments may have been missed, primarily because many instruments are in the gray literature and are not the subject of research papers. It is also possible that the purposive search strategy introduced bias towards data from known researchers in the field. We excluded duplicate surveys (e.g., WHO indicator surveys), as the purpose was to determine the variety of surveys used and not count the frequency of survey use. Furthermore, the reliance on the gray literature means that some instruments may not have undergone rigorous peer review, potentially affecting the reliability and validity of the information. While we extracted the main outcome measure and associated variables (e.g., type of antibiotic, strength, and source of supply) for each data collection instrument, we did not extract the individual questions or compare questions across instruments.

We did not undertake a formal assessment of the validity or reliability of the survey instruments. The major risk regarding the validity of surveys aiming to identify antimicrobial use relates to the accuracy of identification of the relevant medicines. Methods such as interviewer verification of an antimicrobial via visual inspection of the medicine can be used to address this, which can be built into the protocols for household and exit interviews. As discussed above, structured forms to avoid typographical or data entry errors, and short recall periods to avoid memory bias are also methods that can be used to increase the validity of the data.

The results in this paper predominantly relate to paper-based surveys, although, as discussed above, electronic data capture in LMICs is developing as a method for national monitoring of antimicrobials. Electronic data capture will enable more comprehensive assessment of antimicrobial utilization as assessed via prescription or dispensing records, but these methods do not always provide information on use by the patient. Triangulation of results from electronic records and patient- or health professional-focused survey instruments could be used to provide comprehensive assessment of antimicrobial use for the purposes of antimicrobial surveillance.

### 4.5. Implications for Future Research and Policy

The starting point for routine data capture on antimicrobial use in primary care could be to build upon the LMIC national antimicrobial data for GLASS-AMU. Up to 2025, national data have been reported as aggregated for hospitals and primary care in LMICs. The next step would be to disaggregate data to enable analysis of antimicrobial use in primary care and design targeted interventions. This would involve mapping data sources that allow for disaggregation and ensuring that the data are reliable and available. Similarly, countries currently have only the WHO national target of 70% of Access antibiotics [6]. Since most antibiotic use occurs in the community, it is essential to define more general and disease-specific indicators and targets for antimicrobial use in primary care. This ensures that measures can effectively incorporate these parameters for data analysis and action. Harmonizing AMU survey instruments under GLASS-AMU is essential for data comparability and policy relevance across LMICs. Core elements include standardized antimicrobial coding (e.g., International Nonproprietary Names—INNs—and Anatomical Therapeutic Chemical classification system/Defined Daily Dose—ATC/DDD system), indication categories (e.g., International Classification of Diseases—ICD 11), prescription status, payment modality, and basic demographics. As these surveys measure incidence or prevalence rather than consumption volume, alternative indicators should be reported, such as AMU events per 100,000 population or healthcare visits and the proportion of events involving antimicrobial use.

Integrating digital systems for medicine use surveillance in LMICs should follow a phased approach, starting with electronic dispensing records in public hospitals to capture ward- and patient-level data. These systems, installed in hospital pharmacies, can distinguish outpatient from inpatient use and feed into a central database for national analysis. Expansion could later include primary care and community pharmacies. In parallel, monitoring can be embedded in national health insurance schemes, with digitalization either centralized or implemented at the point of care. These databases should capture key variables to enable standardized indicators, such as prescriptions per 100,000 inhabitants, days of treatment (DOT), or defined daily doses (DDD), supporting surveillance and stewardship efforts.

## 5. Conclusions

Overall, our findings reveal a fragmented landscape of antimicrobial use measurement in LMIC primary care settings. While a wide range of instruments exist, they vary considerably in scope, structure, and reporting formats, limiting their comparability and utility for stewardship efforts. The limited integration of the AWaRe classification (represented by only one 2017 study) and the absence of implementation protocols further constrain their potential to inform targeted interventions. These insights underscore the need for harmonized tools and standardized indicators to support more effective monitoring and policy action. Aligning survey instruments with the GLASS-AMU framework and enabling disaggregated data collection, particularly for primary care, will be critical to improving data quality and relevance. Moreover, integrating digital surveillance systems and linking them to national databases can facilitate routine monitoring and support the development of robust indicators for stewardship and access.

This synthesis points to a number of options that could be considered going forward.

First, revise and expand survey protocols to include data on a broader range of medicines beyond antibiotics. This will generate more comprehensive insights into medicine use and help identify gaps and opportunities for health system improvement. Coordinate with national survey teams and stakeholders to align indicators and avoid duplication, enabling integrated data collection across therapeutic areas. Given WHO’s 2024 Access target, the inclusion of AWaRe-related variables should be made an explicit screening criterion in future surveys to ensure alignment with global stewardship priorities.

Second, incorporate electronic data capture systems into antimicrobial use monitoring protocols in LMICs. This includes identifying existing digital platforms, embedding relevant indicators, and training health workers to input and analyze data. Doing so will streamline data collection, improve timeliness, and enhance the quality of antimicrobial use metrics.

Third, develop and deploy standardized implementation protocols and analytic tools to support consistent data collection and interpretation. This involves designing user-friendly templates, piloting them in selected facilities, and building capacity among local teams to use these tools effectively for decision-making.

Fourth, use these tools to assess the appropriateness of antimicrobial use by comparing prescribing practices against recommended treatments in PHC, such as those outlined in the AWaRe antibiotic book. This requires linking prescription data to clinical guidelines, flagging deviations, and providing feedback to prescribers to improve stewardship.

Overall, establish a national framework to map existing data sources, integrate surveys and surveillance systems, and generate disaggregated data on medicine use in primary care. Countries should use this evidence to inform policy, target quality improvement efforts, and strengthen AMR control strategies.

## Figures and Tables

**Table 1:** List of websites of health agencies screened and research networks consulted for this review.

International Society for Improving Use of Medicines (ISIUM)	www.isium.org (accessed on 10 April 2024)
International Pharmaceutical Federation (FIP)	www.fip.org (accessed on 10 April 2024)
Medicines Utilisation Research In Africa (MURIA) network	https://muria.mandela.ac.za/ (accessed on 10 April 2024)
REDCIMLAC (Red de Centros de Información de Medicamentos de Latinoamérica y el Caribe	www.redcimlac.org (accessed on 10 April 2024)
London School of Hygiene and Tropical Medicine (LSHTM) Antimicrobial Resistance Centre	www.lshtm.ac.uk (accessed on 10 April 2024)
ReACT, action on antibiotic resistance	www.reactgroup.org (accessed on 10 April 2024)
Mahidol Oxford Tropical Medicine Research Unit, Thailand (MORU)	www.tropmedres.ac (accessed on 10 April 2024)
HiTAP (Health Information and Technology Assessment Program, Thailand)	www.hitap.net (accessed on 10 April 2024)
Management Sciences for Health	www.msh.org (accessed on 10 April 2024)
WHO Headquarters and Regional Offices	www.who.int (accessed on 10 April 2024)
Bill and Melinda Gates Foundation	www.gatesfoundation.org (accessed on 10 April 2024)
UNICEF	www.unicef.org (accessed on 10 April 2024)
Save the Children	www.savethechildren.org (accessed on 10 April 2024)
USAID	www.usaid.gov (accessed on 10 April 2024)
Antimicrobial use tracker	https://antimicrobialsinsociety.org/antimicrobial-use-tracker/ (accessed on 10 April 2024)
Antibiotic Data to Inform Local Action (ADILA)	https://cnpi-amr.org/research/adila/ (accessed on 10 April 2024)

**Table 2:** Characteristics of identified survey instruments.

Category	Subcategory	Number of Survey Instruments (%)
**By time period**		
Year of conduct	1993–2000	1 (2.4)
	2001–2010	4 (9.5)
	2011–2017	18 (42.9)
	2018–2023 (post-AWaRe)	19 (45.2)
**By geographic region**		
WHO region *	Global (multinational)	7 (16.7)
	Americas	6 (14.3)
	African	7 (16.7)
	Eastern Mediterranean	4 (9.5)
	European	3 (7.1)
	South-East Asian	8 (19.0)
	Western Pacific	7 (16.7)

* The WHO Region of the Americas includes LMICs from Central and South America. The WHO European Region includes LMICs from South-East Europe and Central Asia.

**Table 3:** Household surveys.

Ref.	Target Group	Disease	Where Used	Country	No. and Type of Questions	Main Measure	Reported or Possible Measures
Household survey: Generic							
[15]	Generic	Generic	Home	Jordan	14 closed questions Structured responses; medicine name is text entry	Self-reported, 1-month period prevalence	- Type of medicine - Indication - Treatment duration - Prescription or self-medication - Source of supply - Source of recommendation for use
[16]	Generic	Generic	Home	Multi-country	65 closed questions with structured responses; medicine name is text entry	Self-reported 1-month period prevalence	- Type of medicine - Indication - On prescription - Source of supply - Number of courses - Cost - % taken - % received instructions, written/verbal - % shared after dispensing - % saved for future use
[27] + Sm	Generic	Generic	Electronic survey	Latin America	37 closed questions Structured responses; medicine name is text entry	Self-reported 15-day period prevalence	- Type of medicine - Indication - On prescription - Number of courses - Source of recommendation for use
[17]	Generic	Generic	Generic (home or exit)	Global	3 example surveys 5–15 open questions with unstructured responses	% of illness episodes where antimicrobial used	- Type of medicine - Dose - Duration - Source of supply - Cost
[18]	Generic	Generic	Interviews at bus stations, shopping malls, or health facilities	WHO EUR	15 structured questions plus demographic information	Self-reported use of at least once in last year	- On prescription or self-medication - Reason for use
Household surveys: Child-focused							
[19]	Children under five years	Generic	Home Illness self-report diary	India	30 questions Majority open questions with unstructured responses	% of illness episodes where antimicrobial used	- Type of medicine - Indication - Dose - Prescribed or over the counter - Source of supply - Source of recommendation for use - Cost
[20] + Sm	Children under 2 years	Generic	Home Biweekly illness report form	Multi-country	11 closed questions Structured responses	Courses per child per year	- Class of medicine - Indication
[21]	Children under 2	Generic	Home	Senegal, Madagascar	35 closed questions Mostly structured responses; medication name is text entry; visual cues provided	Self-reported 3-month period prevalence	- Type of medicine - Indication - Dose - Duration - On prescription - Source of supply - Source of recommendation for use - % taken - % shared - % saved for future use
[22]	Children under 5	Diarrhea or acute respiratory tract infection	Home	Uganda	22 closed questions Mostly structured responses; medication name is text entry	Self-reported 2-week period prevalence	- Type of medicine - Indication - Dose - Duration - On prescription - Source of supply - Source of recommendation for use
Household surveys: Disease-focused							
[23]	Children <5 years	Cough	Household	China	30 closed questions with structured responses	Self-reported 1-month period prevalence	- Type of medicine - Source of supply - On prescription
[24]	Generic	Sneeze/cough; diarrhea, gastroenteritis; urethritis	Home	China	62 questions Structured responses	Self-reported 3- or 12-month period	- Indication - Number of antimicrobials - IV antimicrobials - Not on prescription - Source of supply - % using left over antimicrobials
[25]	Generic	Sneeze/cough; diarrhea and gastroenteritis; urethritis	Home	China	68 questions Structured responses	Almost identical to [23]	Refer to [23]
Household surveys: One Health							
[26]	Generic	Generic	Home	China	>100 questions Majority structured responses; includes animal contact	Self-reported One-week, one-month period prevalence	- Type - Source of supply - IV antimicrobials

Sm—Supplementary Materials.

**Table 4:** Exit interviews.

Ref.	Target Group	Disease	Where Used	Country	Number and Type of Questions	Main Measure	Reported or Possible Measures
Exit interviews: Generic							
[27] + Sm	Generic	Generic	Pharmacy	Latin America	37 closed questions with structured responses; medicine name is text entry	Point prevalence	- Type of medicine - Indication - On prescription - Dose - Duration - Source of recommendation for use - Cost
[28] + Sm	Generic	Generic	Public and private facilities: GPs, retail pharmacy shops	India	16 closed questions with unstructured responses	Point prevalence	- Type of medicine - Indication - Dose and duration - Purchased over the counter or dispensed
[16]	Generic	Generic	Medicine suppliers	Multi-country	15 closed questions with structured responses; medicine name is text entry	Point prevalence	- Type of medicine - Indication - Dose and duration - On prescription - Written and verbal instructions
[29]	Generic	Generic		Vietnam	Same as [14]	Same as [16]	- Number of antimicrobial encounters - Number of DDDs supplied - Number of treatment days (DOTs) with antimicrobials - Stratified by AWaRe
[30]	Generic	Generic	Outpatients	Haiti	7 questions Open ended questions; unstructured responses	Self-reported two-week prevalence of self-medicated use	- Limited to self-prescribed antimicrobials only - Type of medicine - Indication - Dose - Duration - Source of supply
[31] + Sm	Generic	Generic	Pharmacies	Latin America	14 closed questions Structured and unstructured responses	Point prevalence Self-reported six-month prevalence	- Type of medicine - Indication - On prescription - Source of recommendation for use - Quantity
[27] + Sm	Generic	Generic	Pharmacy	Latin America	37 closed questions with structured responses; medicine name is text entry	Point prevalence	- Type of medicine - Indication - On prescription - Dose - Duration - Source of recommendation for use - Cost
[32]	Generic	Generic	Primary health facility (post visit at home)	Tajikistan	64 closed questions with structured responses	% of encounters where antimicrobial prescribed	Possible to stratify measure by source, demographics, and indication
Exit interviews: Disease-focused							
[33]	Generic	Acute respiratory illness	Primary care clinics	Kenya	Semi-structured, open-ended; unstructured responses	Point prevalence	- Type of medicine - Indication
[34]	Adults (14 years and over)	Gastroenteritis	Primary care facilities	Nigeria	14 closed questions with structured responses; medicine name is text entry	Point prevalence	- Type of medicine - Indication - Duration - Source of supply
[35] + Sm	Generic	Acute diarrhea	Public and private facilities: GPs, retail pharmacy shops	India	16 closed questions Unstructured responses	Point prevalence	- Type of medicine - Indication - Dose and duration - Purchased over the counter or dispensed
[36] + Sm	Generic	Acute respiratory illness/upper respiratory illness	Public and private facilities: GPs, retail pharmacy shops	India	16 closed questions Unstructured responses.	Point prevalence	- Type of medicine - Indication - Dose and duration - Purchased over the counter or dispensed

Sm—Supplementary Materials.

**Table 5:** Pharmacy audit surveys.

Ref.	Target Group	Disease	Where Used	Country	Number and Type of Questions	Main Measure	Reported or Possible Measures
Pharmacy records							
[37]	Generic	Generic	Pharmacies	Egypt	~20 questions 3 different forms dependent on source of request Structured and unstructured responses.	Antimicrobial prescriptions are the denominator	- Type of medicine - Indication - Dose - Duration - Source of request - Cost
[38]	Generic	Generic	Pharmacies	Europe and Central Asia	13 questions Structured and unstructured responses	Antimicrobial prescriptions are the denominator	- Type of medicine - Dose - Duration - Indication - Prescription or over the counter
[39]	Generic	Diarrhea	Primary Care	India	8 questions Structured and unstructured responses	% of encounters where antimicrobial prescribed	Limited to antimicrobial yes or no

**Table 6:** Physician audit surveys.

Ref.	Target Group	Disease	Where Used	Country	Type of Questions	Main Measure	Reported or Possible Measures
Physician records: Generic							
[40]	Generic	Generic	Public or private primary health care facilities	Philippines	Case report form, unstructured text Summary report form	Antimicrobial prescriptions are the denominator	- Type of medicine - Indication - Dose - Duration
[41]	Generic	Generic	Public or private primary care	Malaysia	Case report form: unstructured text Demographics, reason for encounter, diagnoses, and interventions	% of encounters where antimicrobial prescribed	- Type of medicine - Indication - Dose - Duration
[42]	Generic	Generic	Outpatient clinics at hospitals	India	Semi-structured case report form	% of encounters where antimicrobial prescribed	- Type of medicine - Indication - Duration
[43]	Generic	Generic	Health Facility	Generic	Case report form Unstructured Demographics, reason for encounter, pharmaceutical treatments	% of encounters where antimicrobial prescribed	- Type of medicine - Indication
[44] + Sm	Generic	Generic	Health Facility	India	Case report form Unstructured Demographics, reason for encounter, pharmaceutical treatments	% of encounters where antimicrobial prescribed	- Type of medicine - Indication - Duration
[45]	Generic	Generic	Health Facility	Global	Case report form Structured and unstructured Medicines: unstructured	% of encounters where antimicrobial prescribed	- Type of medicine - Indication - Duration
[46]	Generic	Generic	Health Facility	Egypt	Case report from Structured Medicine names: unstructured	% of encounters where antimicrobial prescribed	- Type of medicine - Indication - Duration
[47] + Sm	Generic	Generic	Health Facility	Sudan	Case report form Unstructured	% of encounters where antimicrobial prescribed	- Type of medicine - Indication - Duration
Generic records: Disease-focused							
[48] + Sm	Generic	Upper respiratory tract infection	Health Facility	South East Asia	Unstructured text	% of encounters where antimicrobial prescribed	- Type of medicine - Indication
[49]	Children under 5y	Diarrhea, pneumonia, acute respiratory infection	Health Facilities	Multi-country	Structured case report form Use of medicine: yes/no	% of encounters where antimicrobial prescribed	Did not collect antimicrobial type
[50]	Generic	URTI	Primary care	Latin America	Structured Demographics, reason for encounter, diagnoses, and interventions	% of encounters where antimicrobial prescribed	- Type (class) of medicine - Indication

Sm—Supplementary Materials.

**Table 7:** Drug store and informal health care worker surveys.

Ref.	Target Group	Disease	Where Used	Country	Number and Type of Questions	Main Measure	Reported or Possible Measures
Corner stores (in stock surveys only)							
[51]	Generic	Generic	Drug store	Uganda	89 questions Structured responses	Limited to stock of antimicrobials; no volume measures	- 5 types of antimicrobials only
[52]	Generic	Generic	Corner store	Guatemala	15 questions—dependent on number of antimicrobials sold Structured responses	Limited to stock of antimicrobials; no volume measures	- Type of medicine - Strength - Formulation - Price
Informal health care providers							
[53]	Generic	Generic	Informal health care provider practice	India	12 questions Unstructured response	% of antimicrobials as a proportion of total prescriptions	- Type of medicine - Indication - Dose - Duration

**Table 8:** Bulk sales data.

Ref.	Data Source	Scale	Country	Copyright	Source	Method	Main Measure
[54,55]	Procurement data for the public health sector	National	South Africa	Permission requested	Website	Time series	DDD/1000/day

## Data Availability

The original contributions presented in this study are included in the article/Supplementary Materials. Further inquiries can be directed to the corresponding authors.

## Supplementary Materials

The following supporting information can be downloaded at https://www.mdpi.com/article/10.3390/antibiotics14111159/s1. Search history, Table S1: Antimicrobial measurement protocols; Table S2: Medicine use protocols, identified protocols. Refs. [20,27,28,31,35,36,44,47,48] are cited in the Supplementary Materials.

## Abbreviations

The following abbreviations are used in this manuscript:

**Abbreviation**	**Definition**
ADILA	Antibiotic Data to Inform Local Action
AMR	Antimicrobial resistance
AMS	Antimicrobial stewardship
AMU	Antimicrobial use
AWaRe	Access, Watch, and Reserve (WHO antibiotic classification)
DDD	Defined daily dose
FIP	International Pharmaceutical Federation
GLASS	Global Antimicrobial Resistance and Use Surveillance System
GLASS-AMU	GLASS Antimicrobial Use module
HICs	High-income countries
HiTAP	Health Information and Technology Assessment Program, Thailand
ISIUM	International Society for Improving Use of Medicines
LMICs	Low- and middle-income countries
LSHTM	London School of Hygiene and Tropical Medicine
MORU	Mahidol Oxford Tropical Medicine Research Unit, Thailand
MURIA	Medicines Utilisation Research In Africa network
PHC	Primary health care
REDCIMLAC	Red de Centros de Información de Medicamentos de Latinoamérica y el Caribe
UNICEF	United Nations International Children’s Emergency Fund
USAID	United States Agency for International Development
WHO	World Health Organization
