# Supplementary material for: Inventory of Survey Instruments for Monitoring Antimicrobial Use in Primary Care Settings in Low- and Middle-Income Countries: A Narrative Review
Source: Antibiotics (Basel). 2025 Nov 15;14(11):1159. doi: 10.3390/antibiotics14111159 (PMC12649748; doi:10.3390/antibiotics14111159)
Supplement: Supplementary file 1 [file antibiotics-14-01159-s001.zip › antibiotics-3919675-supplementary.pdf]

Supplementary file

Search history

Ovid MEDLINE(R) ALL <1946 to September 09, 2022>

1) (afghan or afghans or afghani or albanian? algerian? or american samoan? or angolan? or antiguan? or barbudan? or argentine? or argentinian? or argentinean? or armenian? or aruban? or azerbaijani? or bahraini? or bangladeshi? or bangalees or bajan? or belarusian? or byelorussian? or belizean? or beninese? or bhutanese or bolivian? or bosnian? or botswana or batswana or brazilian? or brasilian? or bulgarian? or burkinabe or burkinese or burundian? or cape verdean? or cabo verdean? or cambodian? or khmer or cameroonian? or central african? or chadian? or chilean? or chinese or colombian? or comorian? or congolese or costa rican? or ivorian? or croatian? or cuban? or djiboutian? or dominican? or ecuadorian? or egyptian? or salvadoran? or equatorial guinean? or equatoguinean? or eritrean? or estonian? or swazi? or swati? or ethiopian? or fijian or gabonese or gabonaise or gambian? or georgian? or ghanaian? or gibraltarian? or grenadian? or guamanian? or guatemalan? or guinean? or bissau guinean? or guyanese or haitian? or honduran? or hungarian? or indian? or indonesian? or iranian? or iraqian? or iraqi? or manx or jamaican? or jordanian? or kazakhstani? or kenyan? or kirabati or kirabatian? or north korean? or korean? or kosovar? or kosovan? or kyrgyz* or lao or laotian? or lebanese or lesothan? or lesothonian? or mosotho or basotho or liberian? or libyan? or lithuanian? or macanese or macedonian? or malagasy or madagascan? or malawian? or malaysian? or maldivian? or malian? or marshallese? or mauritanian? or mauritian? or mexican? or micronesian? or moldovan? or mongolian? or mongol or montenegrin? or moroccan? or mozambican? or burmese or myanma or namibian? or nauruan? or nepali or nepalese or netherlands antillean? or nicaraguan? or nigerien? or nigerian? or northern mariana islander? or mariana? or omani? or pakistani? or palauan? or panamanian? or papua new guinean? or paraguayan? or peruvian? or philippine? or philipine? or phillipine? or phillippine? or filipino? or filipina? or puerto rican? or romanian? or russian? or soviet people or soviet population or rwandan? or rwandese or ruandan? or ruandese or samoan? or sao tomean? or santomean? or saudi arabian? or saudi? or senegalese or serbian? or montenegrin? or seychellois or seychelloise? or sierra leonean? or slovak? or slovene? or solomon islander? or somali? or south african? or south sudanese or sri lankan? or ceylonese or kittitian? or nevisian? or saint lucian? or vincentian? or sudanese or surinamese? or syrian? or tajik? or tajikistani? or tanzanian? or tanganyikan? or thai or timorese? or togolese or tongan? or trinidadian? or tobagonian? or tunisian? or turk? or turkish or turkmen? or tuvaluan? or ugandan? or ukrainian? or uruguayan? or uzbek? or vanuatu* or venezuelan? or vietnamese or yemeni? or yemenite? or yemenese or zambian? or zimbabwean?).ti,ab,sh,kf.913794

2) (afghanistan or albania or algeria or american samoa or angola or "antigua and barbuda" or antigua or barbuda or argentina or armenia or armenian or aruba or azerbaijan or bahrain or bangladesh or barbados or republic of belarus or belarus or byelarus or belorussia or byelorussian or belize or british honduras or benin or dahomey or bhutan or bolivia or "bosnia and herzegovina" or bosnia or herzegovina or botswana or bechuanaland or brazil or brasil or bulgaria or burkina faso or burkina fasso or upper volta or burundi or urundi or cabo verde or cape verde or cambodia or kampuchea or khmer republic or cameroon or cameron or cameroun or central african republic or ubangi shari or chad or chile or china or colombia or comoros or comoro islands or iles comores or mayotte or

democratic republic of the congo or democratic republic congo or congo or zaire or costa rica or "cote d'ivoire" or "cote d' ivoire" or cote divoire or cote d ivoire or ivory coast or croatia or cuba or djibouti or french somaliland or dominica or dominican republic or ecuador or egypt or united arab republic or el salvador or equatorial guinea or spanish guinea or eritrea or estonia or eswatini or swaziland or ethiopia or fiji or gabon or gabonese republic or gambia or "georgia (republic)" or georgian or ghana or gold coast or gibraltar or grenada or guam or guatemala or guinea or guinea bissau or guyana or british guiana or haiti or hispaniola or honduras or hungary or india or indonesia or timor or iran or iraq or isle of man or jamaica or jordan or kazakhstan or kazakh or kenya or "democratic people's republic of korea" or republic of korea or north korea or kosovo or kyrgyzstan or kirghizia or kirgizstan or kyrgyz republic or kirghiz or laos or lao pdr or "lao people's democratic republic" or latvia or lebanon or lebanese republic or lesotho or basutoland or liberia or libya or libyan arab jamahiriya or lithuania or macau or macao or republic of north macedonia or macedonia or madagascar or malagasy republic or malawi or nyasaland or malaysia or malay federation or malaya federation or maldives or indian ocean islands or indian ocean or mali or micronesia or federated states of micronesia or kiribati or marshall islands or nauru or northern mariana islands or palau or tuvalu or mauritania or mauritius or mexico or moldova or moldovian or mongolia or montenegro or morocco or ifni or mozambique or portuguese east africa or myanmar or burma or namibia or nepal or netherlands antilles or nicaragua or niger or nigeria or oman or muscat or pakistan or panama or papua new guinea or new guinea or paraguay or peru or philippines or philipines or phillipines or phillippines or puerto rico or romania or russia or russian federation or ussr or soviet union or union of soviet socialist republics or rwanda or ruanda or samoa or pacific islands or polynesia or samoan islands or navigator island or navigator islands or "sao tome and principe" or saudi arabia or senegal or serbia or seychelles or sierra leone or melanesia or solomon island or solomon islands or norfolk island or norfolk islands or somalia or south africa or south sudan or sri lanka or ceylon or "saint kitts and nevis" or "st. kitts and nevis" or saint lucia or "st. lucia" or "saint vincent and the grenadines" or saint vincent or "st. vincent" or grenadines or sudan or suriname or surinam or dutch guiana or netherlands guiana or syria or syrian arab republic or tajikistan or tadjikistan or tadzhikistan or tadzhik or tanzania or tanganyika or thailand or siam or timor leste or east timor or togo or togolese republic or tonga or "trinidad and tobago" or trinidad or tobago or tunisia or turkey or turkmenistan or turkmen or uganda or ukraine or uruguay or uzbekistan or uzbek or vanuatu or new hebrides or venezuela or vietnam or viet nam or middle east or west bank or gaza or palestine or yemen or zambia or zimbabwe or northern rhodesia or global south or africa south of the sahara or sub-saharan africa or subsaharan africa or africa, central or central africa or africa, northern or north africa or northern africa or magreb or maghrib or sahara or africa, southern or southern africa or africa, eastern or east africa or eastern africa or africa, western or west africa or western africa or west indies or indian ocean islands or caribbean or central america or latin america or "south and central america" or south america or asia, central or central asia or asia, northern or north asia or northern asia or asia, southeastern or southeastern asia or south eastern asia or southeast asia or south east asia or asia, western or western asia or europe, eastern or east europe or eastern europe or developing country or developing countries or developing nation? or developing population? or developing world or less developed countr* or less developed nation? or less developed population? or less developed world or lesser developed countr* or lesser developed nation? or lesser developed population? or lesser developed world or under developed countr* or under developed nation? or under developed population? or under developed world or underdeveloped countr* or underdeveloped nation? or underdeveloped population? or underdeveloped world or

middle-incomecountr* or middle-incomenation? or mid population? or lower income countr* or lower income nation? or lower income population? or underserved countr* or underserved nation? or underserved population? or underserved world or under served countr* or under served nation? or under served population? or under served world or deprived countr* or deprived nation? or deprived population? or deprived world or poor countr* or poor nation? or poor population? or poor world or poorer countr* or poorer nation? or poorer population? or poorer world or developing econom* or less developed econom* or lesser developed econom* or under developed econom* or underdeveloped econom* or middle-incomeeconom* or low income econom* or lower income econom* or low gdp or low gnp or low gross domestic or low gross national or lower gdp or lower gnp or lower gross domestic or lower gross national or lmic or lmics or third world or lami countr* or transitional countr* or emerging economies or emerging nation?).ti,ab,sh,kf. 2088190

3) 1 or 2 2477368

4) (antimicrobial or antibiotic or antibacterial or anti-bacterial or anti-microbial).af.707102

5) (primary health or primary care or community care or health facility or family practice or general practice or general practitioner or physician).af. 704348

6) (drug seller or drug retailer or corner store or drug outlet or pharmacy or pharmacist or informal health worker).af. 637268

7) 5 or 6 1321597

8) (drug utilization or drug utilisation or practice pattern or overuse or underuse or consumption or misuse or inappropriate or overprescr* or underprescr* or over-prescr* or under-prescr* or over-use or under-use).af. 567674

9) 3 and 4 and 7 and 8 1297

10) (questionnaire or survey or protocol).af.1431823

11) 9 and 10 364

12) (defined daily dose or DDD 1000 day).af. 1427

13) 9 and 12 77

14) ((questionnaire or survey or protocol) not hospital).af. 1021315

15) ((questionnaire or survey or protocol) not inpatient).af. 1418706

16) 9 and 15 356

TABLE S1: ANTIMICROBIAL MEASUREMENT PROTOCOLS

Author	Title	Survey types included	Country
Wertheim et al., 2017 (16) protocol link	Community-level antibiotic access and use (ABACUS) in low and middle-income countries	Household survey Exit Interviews	Multi-country
Figueras 2017 (31)	Survey on the use of antibiotics in pharmacies	Household Survey Exit Interviews	Latin America
Pan American Health Organization 2005 (27)	Estimation of the use, quality and cost of antibiotics	Household survey Exit Interviews	Latin America
WHO Regional Office for Europe 2022 (38) protocol link	Antimicrobials supplied in community pharmacies in eastern Europe and central Asia in the early phases of the COVID-19 pandemic	Health Facility Practice Audit: Pharmacy data capture form. Electronic data capture form. Electronic claims data.	Multi-country
Ministry of Health Burkina Faso 2017 (56,70)	Protocol and Report of the consumption of antibiotics in the pharmaceutical offices of the city of Ouagadougou.	Health Facility Practice Audit: Pharmacy; Electronic data capture form.	Burkina Faso
Lubell et al., 2017 (57)	Antibiotic prescription in primary care.	Electronic health records.	Thailand
Robles et al., 2019 (40) protocol link	Manual Procedures for Implementing Antimicrobial Stewardship Primary Care Setting.	Health Facility Practice audit(Public private facilities).	Philippines
Zhao et al., 2019 (58) protocol link	Pathways to optimising antibiotic use rural China identifying key determinants community clinical settings mixed methods study protocol.	Exit interview Household interview Electronic claims data	China
Cook et al.,2025 (46) protocol link	A pilot protocol surveillance infection		

	health facility setting multi-country.		
**One Health Protocols**			
Sun et al., 2018 (24) protocol link	Study protocol for One Health data collections, analyses and intervention of the Sino-Swedish integrated multi-sectoral partnership for antibiotic resistance containment (IMPACT)	Household survey	China
South African Ministry of Health 2017 (52) protocol link	Guidelines on implementation of the antimicrobial strategy in South Africa: One health approach & governance	Bulk sales data	South Africa
**One Health protocol focused on children under 5**			
Stalsby Lundborg et al., 2015 (57) protocol link	Protocol: a 'One health' two-year follow-up, mixed methods study on antibiotic resistance, focusing children under 5 and their environment in rural India	Household survey	India
**Disease based protocols**			
Hopkins et al, 2020 (58) protocol link	Febrile illness Evaluation in a Broad range of Endemicities (FIEBRE): protocol for a multisite prospective observational study of the causes of fever in Africa and Asia	Household survey (Drug Bug Survey)	Multicountry

TABLE S2: MEDICINE USE PROTOCOLS

Author	Title	Survey types included			
Hardon A. et al. 2004 (15) protocol link	Protocol link How to investigate medicine use by consumers Medicine cabinet inventory	Semi structured interviews	Weekly illness recall questionnaire	Family health diary	Medicine cabinet inventory
WHO DAP 1993 (41) protocol link	How to investigate drug use in health facilities	WHO indicators	Physician Case Report Form	ABC analysis	
WHO 2007 (47) protocol link	Protocol link WHO Operational package for assessing, monitoring and evaluating country pharmaceutical situations: guide for coordinators and data collectors	WHO indicators	Condition specific audit form		
WHO South East Asian Regional Office 2016 (46)	Medicines management in health care delivery	WHO indicators	Condition specific audit form		

List of unpublished household survey form

Ref 27: Pan American Health Organization. Estimation of the use, quality and cost of antibiotics. 2005.

Ref 20: Rogawski ET, Platts-Mills JA, Seidman JC, John S, Mahfuz M, Ulak M, et al. Use of antibiotics in children younger than two years in eight countries: a prospective cohort study. Bull World Health Organ. 2017;95(1):49-61.

List of unpublished exit interview survey forms

Ref 28: Kotwani A, Holloway K. Trends in antibiotic use among outpatients in New Delhi, India. BMC Infect Dis. 2011;11(1):99-

Ref 31: Figueras A. Survey on the use of antibiotics in Pharmacies Study Protocol. Catalan Foundation Institute of Pharmacology; 2017.

Ref 27: Pan American Health Organization. Estimation of the use, quality and cost of antibiotics. 2005

Ref 35: Kotwani A, Chaudhury RR, Holloway K. Antibiotic-prescribing practices of primary care prescribers for acute diarrhea in New Delhi, India. Value Health. 2012;15(1 Suppl):S116-9.

Ref 36: Kotwani A, Holloway K. Antibiotic prescribing practice for acute, uncomplicated respiratory tract infections in primary care settings in New Delhi, India. Trop Med Int Health. 2014;19(7):761-8.

List of unpublished physician audit surveys

Ref 44: Mandal P, Asad M, Kayal A, Biswas M. Assessment of use of World Health Organization access, watch, reserve antibiotics and core prescribing indicators in pediatric outpatients in a tertiary care teaching hospital in Eastern India. Perspect Clin Res. 2023;14(2):61-7.

Ref 47: Abdou, E.; Hayder, R.; Salaheldin, M.; Ahmed, A.; Osman, H.; Elhassan, M.; Elamin, A.; Babiker, A. Over-prescription of Watch antibiotics in primary healthcare settings in Sudan: Results from routinely collected prescription data. J. Infect. Dev. Ctries. 2025, 19, 91–97.

Ref 48: South East Asia Regional Office of the World Health Organization. Medicines management in health care delivery. Delhi, India; 2016.
